# A Critically Ill Patient With Central Nervous System Tuberculosis and Negative Initial Workup

**DOI:** 10.3389/fneur.2020.00430

**Published:** 2020-06-12

**Authors:** Adeel S. Zubair, Mark Landreneau, Jens Witsch, Robert K. Fulbright, Anita Huttner, Kevin N. Sheth, David Y. Hwang

**Affiliations:** ^1^Department of Neurology, Yale School of Medicine, New Haven, CT, United States; ^2^Department of Neurology, Stamford Hospital, Stamford, CT, United States; ^3^Department of Radiology, Yale School of Medicine, New Haven, CT, United States; ^4^Department of Pathology, Yale School of Medicine, New Haven, CT, United States; ^5^Division of Neurocritical Care and Emergency Neurology, Yale School of Medicine, New Haven, CT, United States

**Keywords:** CNS TB, tuberculosis, leptomeningeal enhancement, GeneXpert, Meningitis

## Abstract

Empiric anti-tuberculous therapy should not be delayed in patients with a strong clinical suspicion for TB. Because confirmatory TB testing may be difficult to obtain, early and empiric treatment, when there is concern for central nervous system TB, may result in improved outcomes for patients. GeneXpert is currently an area of active research, and the test returns diagnostic results within hours, which would make it the preferred test for investigating TB meningitis.

## Background

Central nervous system (CNS) involvement accounts for about 5–10% of extra-pulmonary tuberculosis (TB) and is present in ~1% of all TB cases (1). The diagnosis of CNS TB can be difficult; a high degree of suspicion is critical. In this report, we describe a patient in the US with severe meningitis of initially unclear etiology who, despite negative TB screening tests, was ultimately found to have tuberculous meningitis upon autopsy.

## Case

The patient was a 62-year-old male with a history of Parkinson's disease and who was admitted to a community hospital with an altered mental status. He was in his usual state of health until 2 weeks prior to presentation when he attended an Albanian cultural festival. A few days afterwards, he started complaining of non-specific upper respiratory symptoms, including cough and sinus congestion, for which he went to an urgent care and received amoxicillin. Despite this treatment, his symptoms continued to worsen over 2 weeks, and he became more lethargic, resulting in his initial presentation to the hospital. The head CT without contrast was unrevealing; a lumbar puncture showed a white blood cell count of 190 with a monocytic predominance (87, 13% polymorphonuclear cells), red blood cell count of 44, glucose of 18, and protein of 235. He was started on vancomycin, ceftriaxone, ampicillin, acyclovir, and dexamethasone. An MRI of the brain with contrast showed leptomeningeal enhancement as well as a left anterior cerebral artery infarct. His lethargy worsened over 3 days, requiring intubation and transfer to our intensive care unit (ICU).

**Figure 1:**
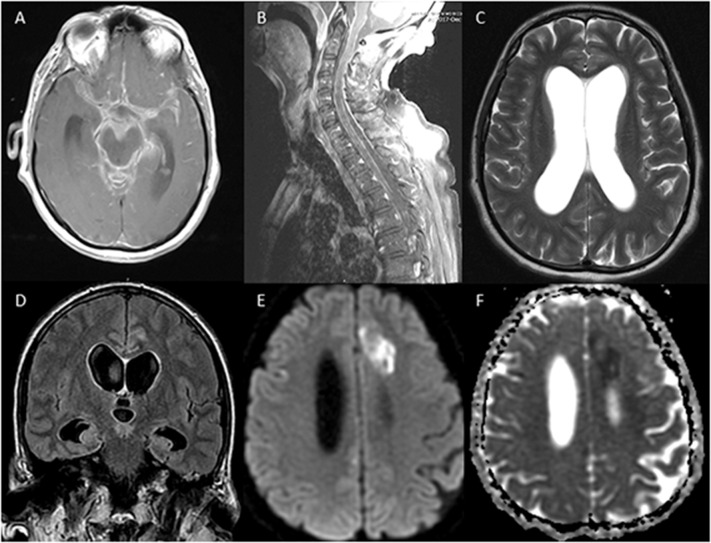
MRI of the brain and of the spine showing evidence of leptomeningeal enhancement **(A,B)**, hydrocephalus **(C,D)**, and acute infarction in the anterior cerebral artery territory **(E,F)**.

On examination upon transfer, the patient was intubated, over-breathing the vent, with no corneal reflex, no gag, no cough, extension in the upper extremities, and triple flexion in the lowers. A repeat MRI of the brain on hospital day 5 demonstrated an evidence of basilar meningitis, interval development of hydrocephalus, persistent leptomeningeal enhancement, most prominent in the bilateral frontal lobes, and the previously seen left anterior cerebral artery territory infarct. An MRI of the total spine also showed leptomeningeal enhancement (Figure 1). CT of the chest/abdomen/pelvis did not show any signs of abscess, adenopathy, or malignancy.

The patient's serum QuantiFERON-TB Gold test was negative when checked originally, as was the repeat 3 days later. The acid-fast bacilli (AFB) cerebrospinal fluid (CSF) smear and culture were also initially reported as negative throughout the hospital course. Serum HIV testing was negative, as was the additional CSF infectious workup. During his ICU admission, the patient developed worsening hydrocephalus and pupillary reactivity despite multiple external ventricular drain placements. MRI of the brain and the spine were done, which showed worsening bilateral supratentorial and infratentorial stroke burden as well as worsening leptomeningeal enhancement around the brain and the spine. The patient progressed to lose all brainstem reflexes over several days and was eventually declared formally brain dead on hospital day 9.

**Figure 2:**
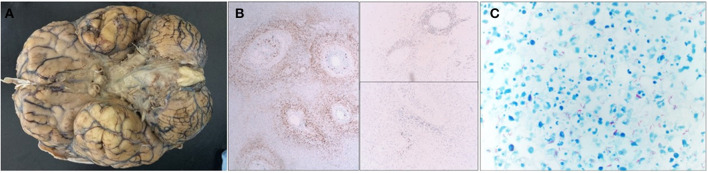
Gross image, immunohistochemical stains, Ziehl–Neelsen histochemical stain. **(A)** The base of the brain is covered by a thick gelatinous subarachnoid exudate, which is particularly pronounced around the midline structures (brain stem and optic chiasm). The brain overall is extremely edematous and stigmata of uncal herniation are seen as well. **(B)** Immunohistochemical stains involving the leptomeninges demonstrate massive inflammation, which is predominately composed of CD3-positive T-lymphocytes, CD68-positive macrophages, and a few CD20-positive B-lymphocytes. **(C)** Ziehl–Neelsen histochemical staining shows an abundance of acid fast bacilli (mycobacteria) in the exudate.

The autopsy showed opacification of the meninges with multiple areas of white exudate collections in the bilateral frontotemporal areas and around the cerebellum and the brainstem. Staining for AFB revealed an abundance of acid-fast bacilli, consistent with *Mycobacterium tuberculosis* (Figure 2). Sections of the cerebrum, basal ganglia, and thalamus showed diffuse edema and foci of subpial necrosis (caseating necrosis), extending into the vessel wall of numerous leptomeningeal and parenchymal vessels and leading to tuberculous endarteritis. At 2 days after the autopsy, the original CSF AFB culture resulted in the growth of *M. tuberculosis* complex.

## Discussion

A diagnosis of extrapulmonary TB can be elusive due to difficulty in obtaining pathologic evidence as cultures take weeks to grow. In a large part because we were biased by this patient's two negative QuantiFERON-TB Gold screening tests, we unfortunately did not send rapid nucleic acid amplification testing [i.e., the (Gene)Xpert MTB/RIF assay] (2) from either sputum or CSF or start empiric anti-tuberculous therapy. Of note is that at least one study has shown that the rate of false-negative serum QuantiFERON-TB Gold testing is around 28.8% in extrapulmonary TB (3). While the sensitivity of sputum Xpert MTB/RIF testing for extrapulmonary TB and the optimal methods of Xpert MTB/RIF testing for CSF samples are areas of active research, the test returns diagnostic results within hours (4), and the World Health Organization has already endorsed Xpert testing as the preferred test for investigating TB meningitis (5). Xpert MTB/RIF has higher sensitivity for TB detection in smear-positive vs. smear-negative patients but nonetheless can be a valuable clinical tool (6).

In addition to facilitating a discussion on the proper methods for TB screening, our case mortality highlights the recommendations expressed in the literature that empiric anti-tuberculous therapy should not be delayed in patients with a strong clinical suspicion for TB (e.g., previous exposure and basilar meningitis on imaging) coupled with CSF findings of low glucose concentration, elevated protein, and lymphocytic pleocytosis (1, 7, 8). Because confirmatory TB testing may be difficult to obtain, early and empiric treatment, when there is concern for CNS TB, may result in improved outcomes for patients.

## Data Availability Statement

All datasets generated for this study are included in the article/supplementary material.

## Ethics Statement

Written informed consent was obtained from the individual(s) for the publication of any potentially identifiable images or data included in this article.

## Author Contributions

AZ and ML contributed to the design and writing of the study and direct patient care. JW and KS contributed to the drafting and revision of the manuscript and direct patient care. RF contributed to the drafting and revision of the manuscript and neuro-imaging review. AH contributed to the drafting and revision of the manuscript and neuro-anatomy review. DH contributed to the design and writing of the study, drafting and revision of the manuscript, and direct patient care.

## Conflict of Interest

JW is a member of the editorial team of “Neurology: Resident & Fellow Section.” The remaining authors declare that the research was conducted in the absence of any commercial or financial relationships that could be construed as a potential conflict of interest.
